# The flexibility of social learning and its conservation implications in mammals and beyond

**DOI:** 10.1098/rstb.2024.0136

**Published:** 2025-05-01

**Authors:** Josh J. Arbon, Neeltje J. Boogert, Neil R. Jordan, Alex Thornton

**Affiliations:** ^1^ School of Biological Sciences, University of Bristol, Bristol BS81TQ, UK; ^2^ Centre for Ecology and Conservation, University of Exeter, Penryn, Cornwall TR109FE, UK; ^3^ Centre for Ecosystem Science, University of New South Wales, Sydney, New South Wales 2052, Australia; ^4^ Taronga Institute of Science and Learning, Taronga Conservation Society Australia, Dubbo, New South Wales 2088, Australia

**Keywords:** conservation, social learning, mammal, flexibility, culture

## Abstract

Conservation strategies seek to ensure that populations persist and are resilient to environmental change. As learning from others can shape the development of skills that help animals survive, reproduce and respond to changing conditions, understanding social learning can be of crucial conservation importance. Research on mammals, with their great diversity of niches and social systems, provides vital evidence that social learning helps animals to communicate, secure mates, avoid predators, forage effectively and navigate through their ecological and social environments. However, these environments are being rapidly altered in the Anthropocene, influencing individuals’ reliance on social learning, the value of learned information, its spread through groups and the stability of socially learned traditions. Here, we review and synthesize this growing body of literature to highlight how understanding the ways in which animals use social learning and deploy it flexibly throughout their lives may enhance conservation programmes. We consider both the potential negative consequences of social learning and the scope for social-learning-driven interventions to generate adaptive responses to the challenges of rapidly changing environments. A greater appreciation and integration of social learning and its flexibility will ultimately promote the effective conservation of mammals and other taxa in our fast-changing world.

This article is part of the theme issue ‘Animal culture: conservation in a changing world’.

## 1. Introduction

Social learning has long been recognized as important for animals to acquire vital foraging, anti-predator and social skills [1,2]. Learning from others is only beneficial if the information acquired will be relevant in the future, so animals use social learning strategies (SLSs) to govern when to rely on social learning (as opposed to individual learning) and whom to learn from [3,4]. However, human impacts on the planet and its climate are increasing environmental variability and disrupting the spatio-temporal autocorrelation of environmental characteristics [5]. If conditions change rapidly and unpredictably, copying previously successful behaviours from others may no longer be adaptive [6]. On the other hand, social learning can also enable the rapid spread of behavioural responses to new challenges, enabling much faster reactions to change than natural selection would permit [6]. These rapid responses can be either beneficial or detrimental depending on context. For instance, socially learned behaviours could promote resilience in the face of human pressure or increase risks of human–wildlife conflict. Understanding social learning processes therefore has crucial conservation implications for both helping to predict how animals will respond to human-induced changes and informing interventions to help conserve or restore natural populations [7,8].

One crucial insight from recent research is that animals can be flexible in their social learning, both in terms of when they use social information and whom they learn from [3,4,9]. This flexibility may have important implications for the development of individual skills and population-level responses to environmental change. Our central aim in this paper is therefore to stimulate discussion and research concerning the practical application of social learning in general, and the flexibility of social learning in particular. We focus primarily on mammals, as this taxonomic group presents a wide range of social systems and life histories and has long been the focus of both social learning research [1,2] and many conservation initiatives [10,11]. Specifically, we focus primarily on ‘other mammals’ as defined by the UN Convention on the Conservation of Migratory Species [12], although we also draw on literature from a broader range of animals where relevant. Other specific mammalian taxa are discussed in detail elsewhere in this issue (primates: [13,14] cetaceans: [15–17], elephants: [18] ungulates: [19]). We first evaluate the conservation value of social learning throughout life, both for animals to learn about their world and for successful intervention by conservation practitioners. We then discuss emerging evidence for flexibility in social learning, how it may impact species’ resilience to change and how a deeper understanding of this flexibility may improve conservation efforts. Our synthesis highlights a current lack of consideration of social learning in many conservation programmes and calls for further generation and implementation of knowledge on social learning when assessing the conservation status of natural populations and when subsequently applying policy.

## 2. Opportunities and costs of learning from (allo)parents

Compared with many other animals, mammals often display long periods of offspring care [20], which provide crucial opportunities for early-life social learning from parents (vertical transmission) or alloparents (oblique transmission). This can enable the rapid development of fundamental skills including species and individual recognition (e.g. recognizing parents; analogous to imprinting [21]), anti-predator behaviour [22,23], foraging skills [24,25] and learning to conform to social group norms [26,27]. Crucially, early life is often a critical period during which learning is particularly likely and often intensified [25,28]. Social learning can begin *in utero*—for example, pregnant rats pass food preferences on to their offspring through the placenta [29]—or shortly after birth through lactation [30]. Rapid learning by juveniles from (allo)parents is widespread within the taxon: young meerkats (*Suricata suricatta*) and smooth-coated otters (*Lutrogale perspicillata*) are more likely to learn new foraging techniques socially than adults [31,32], while black-footed ferrets (*Mustela nigripes*) learn predatory behaviour more effectively as pups when the knowledge is acquired socially than they do when they are older [25]. This social transmission from (allo)parents to juveniles has important conservation implications because it can allow novel behavioural responses to new challenges and opportunities to be transmitted rapidly between generations. The extractive foraging of pinecones (known as pinecone stripping) by black rats (*Rattus rattus*) in Israeli pine plantations is a prime example: experiments have shown that while adults do not learn by observing knowledgeable models, pups readily acquire the skill through interactions with their mother [33,34]. This vertical transmission has resulted in culturally mediated niche expansion, enabling the species to survive in plantations of Jerusalem pine that are otherwise an unsuitable habitat [34]. This provides a powerful example of how understanding the potential for vertical transmission of skills may benefit forecasting of niche expansions, habitat use and adaptability to environmental change.

As well as facilitating the exploitation of new opportunities, however, vertical transmission can also generate human–wildlife conflict. For instance, isotopic and genetic analyses, in combination with statistical models of social transmission, indicate that black bear (*Ursus americanus*) cubs in Yosemite National Park, California, learn bin-foraging habits from their mothers [35]. Similarly, brown bears (*Ursus arctos*) are more likely to demonstrate behaviours that cause conflict with humans, such as foraging on refuse, if their mother also showed these behaviours [36]. Tackling these problematic behaviours is potentially straightforward: if such behaviours are difficult to innovate, breaking the transmission chain should be sufficient to substantially reduce conflict. In practical terms, removing access to these learning opportunities during critical periods for vertical transmission could be an effective conservation tool. In this specific example of bears [35,36], closing picnic areas when knowledgeable females with dependent offspring are in the vicinity should prevent juveniles from learning about these resources from their mothers, thereby avoiding conflict in the future.

Another major vertical transmission challenge for conservation practitioners arises in the context of reintroduction programmes, because captive-reared or rescued animals may not have had the requisite opportunities to learn vital life skills from parents. Many juvenile carnivores, for instance, have especially important periods when they learn hunting skills from their mother [37,38]. In felids such as cheetahs (*Acinonyx jubatus*), mothers provide their cubs with opportunities to handle live prey [38], which may be a means of teaching hunting skills, much like that described in meerkats [39]. Thus, if they are to be successful, reintroductions of captive-reared carnivores may need to recreate learning opportunities that would naturally be provided by adults, for instance by enticing cubs reared in the absence of parents to chase after high-speed prey lures [40]. In other instances, parental models are present, but the correct environment to learn crucial skills is not. Here, creating environments and opportunities in which animals can learn key skills from parents or conspecifics will be crucial to their development. For example, large, naturalistic pre-release pens [41] that enable the observation of (allo)parents interacting with relevant predator and prey cues [23,42] may provide a promising avenue for increasing post-release success. Replacing lost social learning opportunities may be especially important for learning to correctly identify and respond to predators. Indeed, predation of naive individuals is a leading cause of mortality of re-introduced animals [10]. Anti-predator behaviour is known to have a large socially learned component in many species [1,2,43], so harnessing social learning may be a crucial tool [28]. For example, experiments examining pre-release training showed that Tammar wallabies (*Macropus eugenii*) can learn about novel predators by observing the reactions of trained conspecifics [23]. Further, black-tailed prairie dogs (*Cynomys ludovicianus*) exposed to predator models during pre-release training developed more appropriate wary responses if trained in the presence of a knowledgeable adult female compared with those trained alone [22]. Crucially, this training enabled captive-reared juveniles to behave like, and consequently to survive at comparable rates to, wild-reared conspecifics [22]. There is also anecdotal evidence that pre-release training can restore chains of information transmission, enabling surviving reintroduced animals to pass on knowledge to wild-born offspring [44]. However, it remains unclear to what extent survival skills acquired through captive training are subsequently transmitted to the next generation in the wild. Post-release monitoring is, therefore, vital to assess the knowledge (e.g. eating suitable foods, responding correctly to threats) and ultimately fitness (e.g. do they survive and reproduce at comparable rates to wild-born animals?) of released animals and their offspring and enable further refinement of training regimes. Open-access forums such as Conservation Evidence [45] can provide venues through which the outcomes of such training facilitation or interventions can be easily accessed and digested by conservation practitioners (see also [46]).

An additional consideration when training individuals for release is the potential for mismatches between their evolutionary history and their current environment. Many species exhibit predispositions to learn specific types of stimuli: one classic example is in rhesus monkeys (*Macaca mulatta*), that readily learn to fear snakes (a relevant predator) but not flowers (an innocuous stimulus) if they are paired with the sight of conspecifics showing anti-predator responses [47]. However, adaptive responses to evolutionarily relevant threats can also be rapidly lost: northern quolls (*Dasyurus hallucatus*) that were moved to a predator-free offshore island to stop them from consuming poisonous cane toads (*Rhinella marina*) lost their antipredator responses to dingoes (*Canis lupus dingo*) after just 13 generations, spending less time inspecting dingo scent [48]. This presents a problem for reintroduction efforts to areas that have predators. Attempts to re-train quolls to fear dingoes using the pairing of a live predator and an aversive stimulus (electric shock) were unsuccessful [49], demonstrating the dangers of rearing animals in predator-free environments, either in captivity or in areas where predators have been lost and may subsequently return or be reintroduced. This also highlights that including adult social models such as (allo)parents may be key to increasing the efficacy of retraining lost knowledge. However, whether animals can be trained to show appropriate anti-predator responses to novel predators remains unclear. For example, invasive Burmese pythons (*Python molurus bivittatus*) have been linked to the severe decline of many mammal species in the Florida Everglades, resulting in decreases of 99% in raccoon (*Procyon lotor*) and opossum (*Didelphis virginiana*) populations [50], but it is unclear how these mammals respond when encountering these novel threats. Given the widespread success of novel predators introduced by human activity [51] and direct threats posed by humans (e.g. hunting, roadkill), we need to further our understanding of where, when and how anti-predator behaviour can be both acquired and lost [52], and how social transmission can help reduce predation and its impact on vulnerable populations.

## 3. Mismatches and flexibility in early-life models

To understand the adaptive value of social learning throughout life and to harness its conservation value in a changing world, we need to consider environmental mismatches. One way that mismatches could impact social learning is through changes in phenology, where the timing of crucial events in the lifecycle of organisms shifts and becomes misaligned with resource availability. Phenological mismatches are broadly recognized as having large negative effects on animals’ fitness [53], making them highly relevant for conservation. Phenological shifts could, in theory, also have substantial impacts on opportunities to learn information and skills that affect survival and reproduction. However, concrete examples of how these mismatches affect social learning opportunities are lacking and implications for conservation have so far been overlooked. For example, one may hypothesize that if the timing of breeding in predator species shifts (as has been seen in African wild dogs [54]), a mismatch between the breeding of predators and prey may occur. Young predators will therefore have reduced opportunities to learn to hunt small, vulnerable prey socially before the latter develop behavioural and physical defence mechanisms (e.g. horns). This could force inexperienced hunters to pursue more difficult, dangerous prey, with reduced chances of success and increased risks of injury or death. Given the diverse taxa and foraging strategies reported to be impacted by phenological mismatches [55], considering the potential impacts of phenological mismatches on social learning opportunities and subsequent fitness outcomes is important if we are to fully appreciate how changing conditions affect population dynamics and ecosystem resilience.

**Figure 1.**
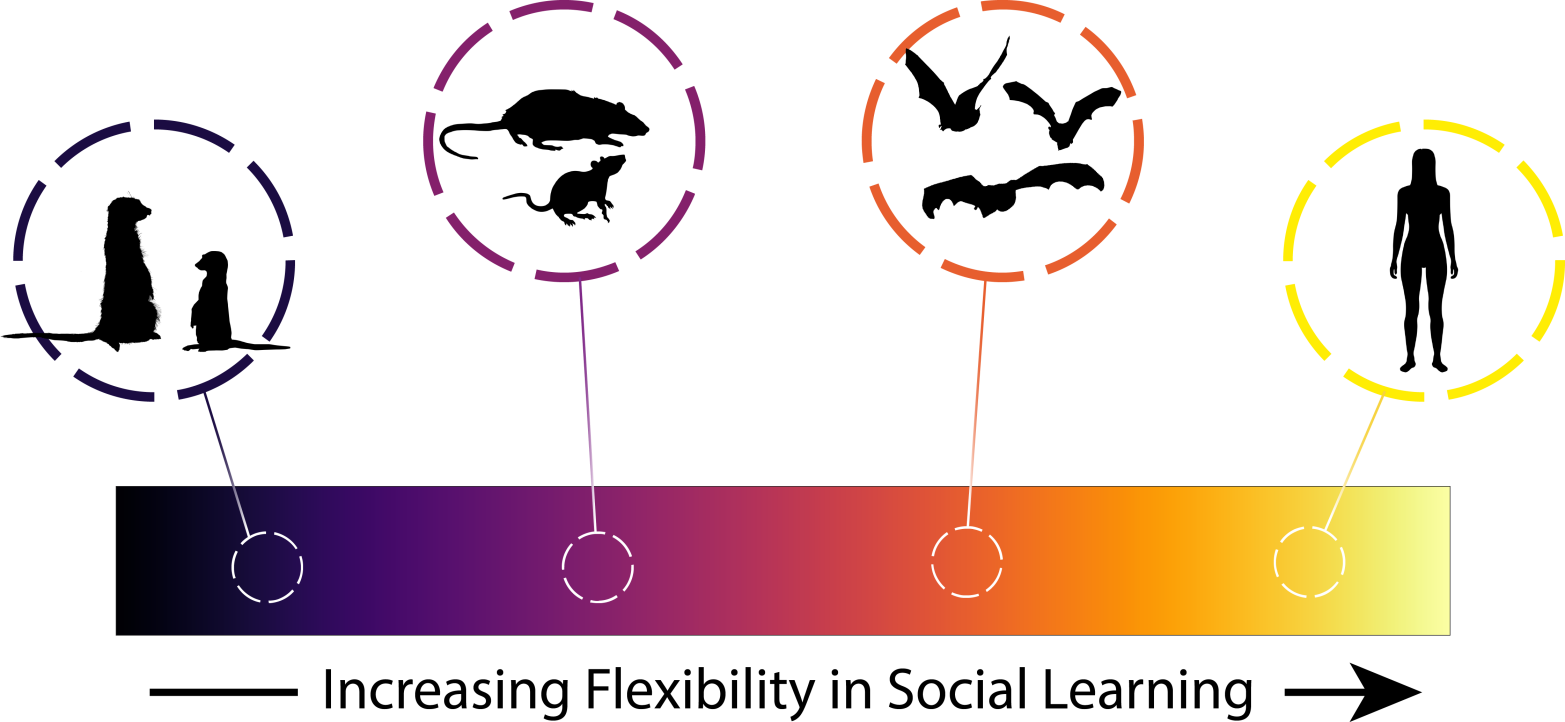
Gradient of flexibility in social learning, adapted from [9], with examples drawn from the ‘other mammals’ taxon, with the exception of humans. The colour gradient from left to right represents increases in the flexibility of social learning. Furthest left represents greater inflexibility, such as in meerkat pups that obligately learn about new foods from adult conspecifics [62]. The next group represents switching of learning models owing to environmental cues (e.g. developmental stress), predicted to be possible in mammalian taxa where young have access to many potential adult models (e.g. rats [63]). More flexible still is learning which models are reliable or preferential to learn from socially , such as in bats [64,65]. Furthest right represents the most flexible, namely *social* learning of SLSs, which is seen potentially only in humans [9].

In addition to losses in social learning opportunities, another way in which mismatches can impact social learning is through a reduction in the reliability of learnt information. Social learning in early life is only adaptive when there is temporal autocorrelation—that is, when the state of the current environment is predictive of the future environment [6]. Changes to the environment may render the acquisition of knowledge through vertical or oblique transmission maladaptive if the behaviour that was optimal for adults is no longer optimal in the environments encountered by offspring. For example, species that show site fidelity—where individuals learn and then faithfully recruit to a location—in roosting (as in many bat species [56,57]), feeding (as in northern elephant seals *Mirounga angustirostris* [58]) and breeding (as in grey seals *Halichoerus grypus* [59]) will be vulnerable to changes in environments [60]. Similarly, there may be mismatches between an individual’s current environment and that in which its parents bred or developed. However, if individuals can show flexibility in the strategies that govern whom they learn from and when [3,4], this may help to counter mismatches between what parents are doing and what is optimal. One clear example of such strategic switching has been demonstrated by zebra finches (*Taeniopygia castanotis*). In this species, juveniles typically follow and learn from their parents, but if exposed to experimentally elevated developmental stress (which could be a cue of poor parental quality), they switch from a ‘learn from parents’ strategy to a ‘learn from non-parents’ strategy [61]. Here, a physiological input served to alter learning models, which could increase the probability of adaptively copying from adults that are currently successful in the environment (figure 1). This may provide important advantages in rapidly changing environments [66]. Although the potential for such stress-mediated switches in learning strategy is as yet untested in mammals, stress is known to impact sociality in diverse species within the taxon. For example, developmental stress alters later-life social relationships in olive baboons (*Papio anubis*) and meerkats [67,68], while exposure to stress later in life affects the formation of relationships in meadow voles (*Microtus pennsylvanicus*) [69], highlighting the potential for analogous switches in learning models.

These considerations of flexibility in the source of animal learning during early life are to become increasingly relevant to conservation efforts with increasing global change. In many species, the young tend to imprint on the first animals they encounter [21]; in evolutionary history, this has almost always been a conspecific. This can lead to inflexibility in social learning that has evolved to canalize learning from adaptive models. For example, meerkat pups are extremely neophobic about food but will sample novel food items if they see conspecific adults eating them. If the same items are presented to them by a human experimenter, however, pups will not touch them [62] (figure 1). Such inflexibility, by eating only what they see conspecific adults eating [62] and relying on adults to teach them to handle difficult prey [39], is adaptive under the relatively stable conditions in which the species evolved. In changing environments, however, such strict dependence on social learning from adult conspecifics could reduce individual innovation and limit the potential to acquire valuable new information from other sources, such as heterospecifics [70]. Many mongoose species, including meerkats, frequently use anti-predator information from other species [71], while multiple bat species attend to heterospecific cues in a foraging context [72,73] (figure 1). Heterospecifics can therefore be another source of useful novel information in changing landscapes, but only if individuals will attend to and learn from them [74].

Conversely, a tendency to learn from interactions with heterospecifics can also generate conservation problems—particularly in instances where young animals learn to attend to people. In changing environments, many young mammals such as koalas (*Phascolarctos cinereus*), raccoons and cheetahs are rescued from the wild with the intention of rehabilitation and release [10,11,75]. However, during rehabilitation, animals can lose fearful responses to humans, often with negative consequences for species that frequently come into conflict with people [10]. Post-release survival of captive-raised cheetahs, for example, was negatively related to their level of human habituation, with 50% of post-release mortality resulting from human persecution [76]. It is therefore important for us to increase our understanding of the potential hazards of animals learning to attend to human caregivers, however well-intentioned, and the potential costs to individuals’ chances of survival and reintegration once rehabilitated and released. Similar challenges exist in conservation-based breed-for-release programmes, with many programmes in other taxa identifying the benefits of imprinting young animals onto models of their own species (e.g. puppet rearing in birds [77]) to ensure that animals develop the correct search images for conspecific social models, as well as reducing conflict with humans [76]. Research on corvids shows that fearful responses towards people can be learnt socially and persist for many years [78,79]. This demonstrates the potential to harness social learning to train animals to show fearful responses to humans and thereby to reduce anthropogenic mortality, although further work is needed to determine whether this is practicable across different taxa. Recent research has also highlighted the benefits of using wild models over their captive counterparts where possible (e.g. in song learning [80]), and given that animals can learn socially from video recordings [81] and robotic models [82], the possibilities to ensure that animals receive appropriate demonstration are continually growing.

## 4. Learning to be flexible

While vertical transmission may provide mammals with many core skills, social learning is by no means restricted to early life and learning from peers can facilitate rapid responses to change [83]. Many species have abundant opportunities to learn from a variety of models throughout their lives and therefore must make adaptive decisions regarding when to learn socially and from whom [3]. These decisions have traditionally been assumed to be underpinned by genetically controlled strategies that evolve through natural selection, but there is growing evidence that the deployment of SLSs is shaped by experience [9,84,85]. One potential strategy is to prioritize social learning when uncertain, as others may have better information. Uncertainty may decline as individuals get older and gain experience, causing them to switch to prioritizing individuals over social information—as seen in the migratory strategies of whooping cranes (*Grus americana*) [86]. The link between uncertainty and propensity to use social over personal information was tested experimentally in fringe-lipped bats (*Trachops cirrhosus*). Bats were individually trained on a novel sound cue that was associated with food either 50% or 100% of the time in the presence of a conspecific feeding on a different, reliable sound cue. Those trained on the randomly rewarded, unreliable cue were more likely to switch and learn the conspecific's novel prey cue compared to those trained on the reliable cue [65] (figure 1). If environmental cues become less reliable, some species will be more likely to switch from individual learning to learning from others, thus effectively buffering against change. Assessing a species' ability to do this is therefore a key conservation priority.

Another route to flexibility is learning whom to learn from. Many described SLSs, such as learning from older or dominant individuals, rely on a characteristic of an individual that is likely to correlate with their experience and therefore ability or knowledge [4,87,88]. These shortcuts prevent animals from needing to assess the performance of many others, which could entail large cognitive and time-budget costs. These same shortcuts can also be exploited by conservation practitioners when attempting to seed behavioural change in a population or prevent maladaptive behaviour from spreading by targeting the removal of specific influential individuals [89]. However, the individuals that provide the best information may change, particularly in increasingly variable environments. Thus, correlations between observable characteristics (e.g. age, dominance) and knowledge or performance may break down, rendering these strategies maladaptive [84]. For instance, in a changing world, animals may need to innovate to overcome novel challenges [90,91]; therefore, since younger individuals can be more innovative [92], relying on learning from older individuals may no longer be the best strategy. The ability to switch learning models to those who are currently successful may therefore be highly valuable.

This premise is broadly captured by the ‘learn from the most successful’ strategy, where animals directly assess the quality of others and learn accordingly. For example, evening bats (*Nycticeius humeralis*), a species that forages from a central roost, were more likely to follow well-fed individuals on foraging trips when they themselves had foraged unsuccessfully, a strategy that resulted in weight gain compared to those that continued to forage alone [64] (figure 1). Strikingly, fruit flies (*Drosophila melanogaster*) provide some of the most compelling evidence for using the success of others as a learning cue: females were more likely to lay eggs on substrates where they had witnessed mated (i.e. successful) females laying as opposed to laying where unmated (i.e. unsuccessful) females laid [93]. Similarly, success-biased learning has been demonstrated in pied flycatchers (*Ficedula hypoleuca*) [94] and is suggested to be used by chimpanzees (*Pan troglodytes*) [95]. The use of these strategies by animals across taxa suggests that abilities to track changing information about whom is best to learn from are widespread and not necessarily cognitively demanding. One step further, there is emerging evidence that animals can learn which demographics of individuals to learn from and thereby update their learning strategies. Adult jackdaws (*Corvus monedula*) were able to learn to tolerate and associate with juveniles to gain food rewards [96], generalizing across the cohort from experience with specific juveniles, despite juveniles previously being ignored as models for social learning [97]. This represents learning of a new strategy: ‘tolerate and attend to juveniles’. Such flexibility could be powerful for animals to rapidly switch whom to learn from if the best exemplars change. An even greater degree of flexibility could arise if individuals can learn new strategies by observing others. Such *social* learning of social learning strategies (figure 1) is currently thought to be specific to humans [9].

An important implication of flexible learning about good models is that there can be feedback with social network structure that have potential knock-on effects for conservation. For example, jackdaws and ring-tailed lemurs (*Lemur catta*) have both been shown to adjust their social relationships based on their assessments of social partner quality, leading to knock-on changes in social network structure [98,99]. Flexible learning-induced changes in social networks can expedite the transfer of information, which could help species buffer against changes in their environment. There are empirical links between behavioural flexibility and both increased success in invading new habitats [90] and reduced extinction risk [91] in birds, where a relationship between increased flexibility and larger brains is suggested as a driver. It remains an outstanding question whether these relationships between behavioural flexibility and species persistence occur in mammals. Investigating behavioural flexibility across taxa is thus an important priority for fundamental and applied science alike.

One potentially underappreciated issue is that the very characteristics that should help species flexibly learn to buffer against human changes can be the same characteristics that can drive animals into conflict with people. Species that can rapidly learn to benefit from a new foraging resource may be able to buffer against the loss of traditional food sources. However, if this new resource is a crop or livestock, this will generate human–wildlife conflict [100]. For example, salmon ladders are structures designed to enable salmonids (*Oncorhynchus* spp.) to migrate across man-made barriers such as dams, but by nature they cause salmonids to gather in a specific location. California sea lions (*Zalophus californianus*) have learned to forage at the bottom of these structures, and this behaviour is socially transmitted [101]. This has resulted in large-scale salmon foraging, thereby jeopardizing the effectiveness of salmon conservation interventions, which in turn has led to the widespread culling of sea lions [101]. In this instance, removing the early adopters of unwanted behaviour through culling was suggested as the key to stopping the spread early and minimizing future costs [101], especially as less severe deterrents were not effective [102]. If sea lions use flexible learning as described above, where innovating sea lions would become more likely to be learned from (e.g. if they display signs of foraging success, such as good condition or scent traces from prey), this could lead to the more rapid adoption of this behaviour and a worsening of the human–wildlife conflict. Furthering our understanding of how learning flexibility may exacerbate these and similar conservation issues will be critical in identifying the scale of potential problems and optimizing subsequent interventions, such as the identification and management of problem individuals [89].

## 5. Evolutionary history, social structure and flexibility

**Figure 2.**
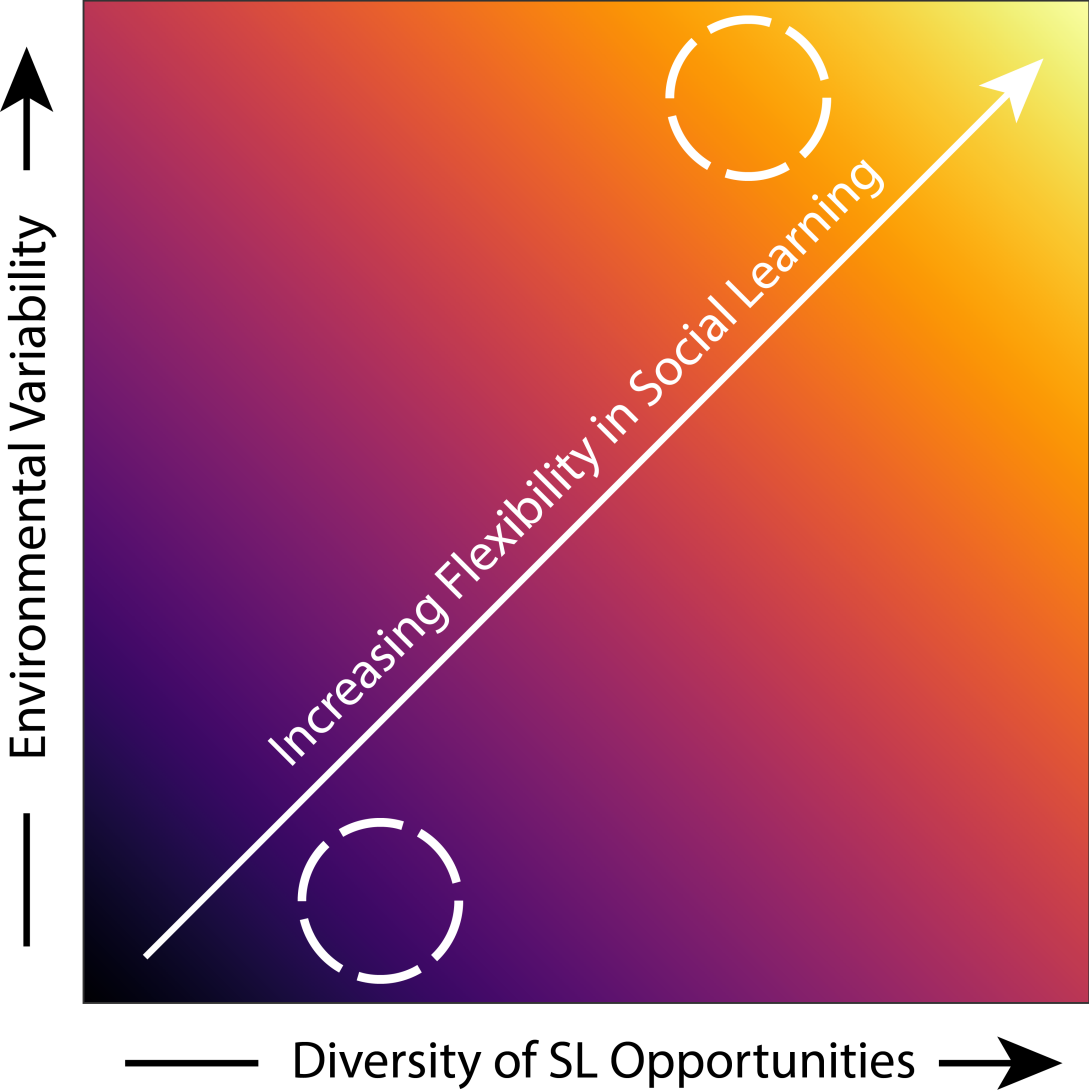
Evolutionary factors are predicted to influence the propensity of species to be flexible in their social learning (SL). Both the variability of the evolutionary environment and the diversity of SL opportunities in the evolutionary social structure are predicted to have a positive influence on the flexibility of SL. In this instance, both factors are assumed to have equal importance and interact additively, but this may not be the case. The colour gradient is the same as in figure 1, where dark is inflexible and light is highly flexible, as highlighted by the diagonal arrow. The circle in the upper right highlights species that may be able to leverage flexibility in SL to cope with anthropogenic change, although these may also be the species that are most likely to come into conflict with humans. The circle in the bottom left represents species that are less likely to be able to use flexible SL to buffer against anthropogenic change. Optimal conservation strategies are therefore likely to be different across the colour gradient.

A major outstanding question of relevance to conservation is ‘in which taxa might we find flexibility in social learning?’. One way to help answer this is to look at the evolutionary history of species and which factors predict behavioural flexibility more generally. In broad terms, greater environmental variation will select for phenotypic plasticity [103] to allow animals to change their behaviour more rapidly to match changes in conditions. Theoretical models predict that at low environmental variability, genetic inheritance is more efficient than social learning, intermediate variability favours social transmission, and at very high variability, the lack of reliability of learning models favours individual, trial-and-error learning [6,84]. In environments that are highly variable—where there is uncertainty in the payoffs of social learning—developmental flexibility in reliance on social learning can be favoured such that experiences of the reliability of social information influence the likelihood of copying others. This adds theoretical support to the assertion that SLSs can themselves be learned [85]. We would predict that if individuals can flexibly adjust their choice of learning models, social learning may be adaptive at greater levels of environmental variability than current theoretical models suggest [6,84,85]. Substantial variability could be selected for individuals to track others’ success and update their SLSs to keep up-to-date with those con- or heterospecifics that are best adapting to changing conditions (figure 2). The specific factors that drive differences in SLSs and flexibility both within and across taxa are important targets for future fundamental and applied research.

Alongside the stability of the environment, social structure is another major component of the evolutionary history of animals that may affect the flexibility of social learning. Differences in social structure affect learning opportunities and, therefore, should influence evolved predispositions to learn flexibly. Solitary mammals may be more likely to rely more heavily on vertical transmission during early life owing to the lack of opportunities to learn socially about the environment in later life. In contrast, species for which many different sources of information are available—such as many bat species that live in fission–fusion societies [104]—may have more access to the information that enables flexibility and greater evolutionary pre-dispositions to use social learning flexibly (figure 2). Human activity often generates concentrated resources—such as at refuse or agricultural sites—where animals aggregate at high densities [105], generating opportunities for social interaction that may not have been present in the species’ historic ecology. Such clustering of animals will provide novel social learning opportunities, both for animals to observe and learn from one another locally, but also for these areas to act as information centres [106]. However, we would expect that in instances where typically solitary species now congregate and social interactions have become more common (e.g. foxes or leopards in urban areas), individuals may not be predisposed to attending to others or be flexible in their social learning [107] owing to their evolutionary history.

The relevance of social structure for social learning in the context of conservation is highlighted by the invasion of cane toads (*Rhinella marina*) in Australia. The success of this human-introduced invader has caused large declines in many species such as Northern quolls, which eat the toxic toads and often die as a result. Conditioned taste aversion through the presentation of toad-flavoured but non-lethal baits has been shown to reduce the likelihood of quolls attacking toads [42]. Witnessing others demonstrate an aversion to a potentially toxic food is known to promote learning in some bird species [108], so in principle, social learning could be explored as an avenue to optimize the spread of aversion to new, potentially toxic or dangerous animals such as cane toads. Further assessment of the mechanisms underlying social learning of aversion will be important for useful implementation [109]; for instance, we must determine whether animals need to observe others showing aversive responses or if aversion can be learnt from their products (e.g. deceased conspecifics or regurgitated toads). Learning from products may be especially important for species such as quolls that are largely solitary and therefore have few opportunities for horizontal learning from conspecifics. We would also expect that typically solitary animals would not be pre-disposed to exhibit flexibility in their social learning (figure 2), further decreasing the likelihood of adaptive aversion spreading. In contrast, gregarious and social species are more likely to have horizontal learning opportunities to exhibit greater flexibility in their social learning. Torresian crows (*Corvus orru*), for instance, have learned to eat only the tongues of the toads, which are less toxic, with circumstantial evidence that this innovation is socially transmitted [110]. We would therefore suggest that any social learning-based interventions on species such as quolls will be more effective when targeting vertical transmission; indeed, reintroduced ‘toad-smart’ quolls survived and appeared to pass on their anti-toad preferences to their offspring, which were subsequently able to survive and reproduce [44]. In these instances, focusing on training mothers may be a more effective approach to facilitate the spread of such adaptive knowledge. Expanding this concept, in species that exhibit strong learning biases or have disproportionately influential individuals, seeding aversive behaviour in such ‘social influencers’ will be the most effective way of rapidly disseminating adaptive behaviour [107]. Finally, in species that demonstrate flexibility in learning and that live in well-connected social structures—such as in bat species where roost sites can act as information centres [106]—there may be less need to target certain individuals or demographics for conservation interventions because individuals demonstrating newly adaptive behaviour may be readily learned from and become more integrated and central in social networks as a result [111].

## 6. Concluding remarks

Social learning remains largely overlooked both as a tool in conservation strategies and a factor that may hamper conservation outcomes. In the face of human-induced rapid environmental change, social learning will be vital in allowing animals to adapt to changing conditions at rates higher than natural selection normally allows. However, factors that can promote flexibility, resilience and persistence can also predispose species to human–wildlife conflict. Given that the consequences of social learning can potentially assist or derail conservation efforts, it is surprising that social learning rarely features prominently, if at all, in conservation planning and actions. Animal rehabilitation, conservation translocation and breed-for-release programmes are common conservation approaches where outcomes may either be harmed by the consequences of inappropriate social learning, such as imprinting on humans, or enhanced by concerted efforts to facilitate the acquisition of beneficial behaviour through social learning. While a few recent conservation efforts or recommended interventions have benefitted from the integration of social-learning-focused approaches [22,77,80], these remain the exception rather than the rule. Research into the flexibility of social learning in particular is an exciting opportunity to further our understanding of natural processes, therefore aiding forecasts of species’ responses to human change and informing the optimization of conservation interventions. Given the historic social learning research conducted and the experimental tractability of many mammalian systems [2], it is perhaps surprising that research into social learning and its use in conservation is somewhat behind that in avian taxa [112]. Optimizing the social learning potential and performance of mammalian systems may be key to generating positive conservation outcomes for non-human animals and our own species alike.

## Data Availability

This article has no additional data.
